# Effects of the Probiotic, *Lactobacillus delbrueckii* subsp. *bulgaricus*, as a Substitute for Antibiotics on the Gastrointestinal Tract Microbiota and Metabolomics Profile of Female Growing-Finishing Pigs

**DOI:** 10.3390/ani12141778

**Published:** 2022-07-11

**Authors:** Jiayuan Mo, Yujie Lu, Shan Jiang, Gang Yan, Tianqi Xing, Di Xu, Yaoyin He, Bingkun Xie, Ganqiu Lan, Baojian Chen, Jing Liang

**Affiliations:** 1 College of Animal Science & Technology, Guangxi University, Nanning 530004, China; gxu_mojiayuan@163.com (J.M.); lu_yujie222@163.com (Y.L.); js591954779@163.com (S.J.); yangang0805@163.com (G.Y.); 2118391051@st.gxu.edu.cn (T.X.); xudi18788704824@163.com (D.X.); gxu_ycyz@163.com (Y.H.); bkxie@163.com (B.X.); gqlan@gxu.edu.cn (G.L.); 2 Guangxi Key Laboratory of Livestock Genetic Improvement, The Animal Husbandry Research Institute of Guangxi Zhuang Autonomous Region, Nanning 530004, China

**Keywords:** gastrointestinal tract, microbiota, metabolites, *Lactobacillus delbrueckii* subsp. *bulgaricus*, co-occurrence network

## Abstract

**Simple Summary:**

*Lactobacillus delbrueckii* subsp. *bulgaricus* (LDB) is an important candidate for antibiotic replacement in pig production. In this study, LDB and antibiotic diets were fed to the LDB and antibiotic groups of female growing-finishing pigs, respectively. 16S rRNA sequencing was used to identify different microbiota. Liquid chromatography-mass spectrometry-based non-targeted metabolomics approaches were used to identify different metabolites. The co-occurrence network of the fecal microbiota and metabolite was analyzed. The results contain information on pig growth performance, microbiota data, metabolite data and co-occurrence networks, supporting the possibility of LDB as an antibiotics replacement in pig production.

**Abstract:**

*Lactobacillus delbrueckii* subsp. *bulgaricus* (LDB) is an approved feed additive on the Chinese ‘Approved Feed Additives’ list. However, the possibility of LDB as an antibiotic replacement remains unclear. Particularly, the effect of LDB on microbiota and metabolites in the gastrointestinal tract (GIT) requires further explanation. This study aimed to identify the microbiota and metabolites present in fecal samples and investigate the relationship between the microbiota and metabolites to evaluate the potential of LDB as an antibiotic replacement in pig production. A total of 42 female growing-finishing pigs were randomly allocated into the antibiotic group (basal diet + 75 mg/kg aureomycin) and LDB (basal diet + 3.0 × 10^9^ cfu/kg LDB) groups. Fecal samples were collected on days 0 and 30. Growth performance was recorded and assessed. 16S rRNA sequencing and liquid chromatography-mass spectrometry-based non-targeted metabolomics approaches were used to analyze the differences in microbiota and metabolites. Associations between the differences were calculated using Spearman correlations with the Benjamini–Hochberg adjustment. The LDB diet had no adverse effect on feed efficiency but slightly enhanced the average daily weight gain and average daily feed intake (*p* > 0.05). The diet supplemented with LDB increased *Lactobacillus* abundance and decreased that of *Prevotellaceae_NK3B31_group* spp. Dietary-supplemented LDB enhanced the concentrations of pyridoxine, tyramine, D-(+)-pyroglutamic acid, hypoxanthine, putrescine and 5-hydroxyindole-3-acetic acid and decreased the lithocholic acid concentration. The *Lactobacillus* networks (*Lactobacillus*, *Peptococcus*, *Ruminococcaceae_UCG-004*, *Escherichia-Shigella*, acetophenone, tyramine, putrescine, N-methylisopelletierine, N1-acetylspermine) and *Prevotellaceae_NK3B31_group* networks (*Prevotellaceae_NK3B31_group*, *Treponema_2*, monolaurin, penciclovir, N-(5-acetamidopentyl)acetamide, glycerol 3-phosphate) were the most important in the LDB effect on pig GIT health in our study. These findings indicate that LDB may regulate GIT function through the *Lactobacillus* and *Prevotellaceae_NK3B31_group* networks. However, our results were restrained to fecal samples of female growing-finishing pigs; gender, growth stages, breeds and other factors should be considered to comprehensively assess LDB as an antibiotic replacement in pig production.

## 1. Introduction

Probiotics are defined as “live strains of strictly selected microorganisms that, when administered in adequate amounts, confer a health benefit to the host” [1]. Probiotics have been widely researched in humans [2], rats [3], chickens [4], cattle [5] and pigs [6]. The most used microorganisms belong to the genera *Lactobacillus* [7], *Bifidobacterium* [8] and *Saccharomyces* [9]. Probiotics in pigs play important roles, such as defending against viral infection [10], enhancing meat quality [11], improving immune function [12] and increasing growth performance [13]. Most importantly, probiotics (especially *Lactobacillus* spp.) [14,15] are used as substitutes for antibiotics in pig production [16]. A previous study showed that *Lactobacillus* enhanced the reproductive performance of sows and the growth performance of weaned piglets [17]. According to a study by Chen et al., *Lactobacillus* can provide some levels of protective effect against porcine epidemic diarrhea virus infections [18]. Tian et al. report that *Lactobacillus* can enhance meat quality by increasing the concentration of inosinic and glutamic acid concentrations, decreasing drip loss and shear force [19]. Moreover, Geng et al. proposed that *Lactobacillus* may promote the immunity of weaned piglets by regulating cytokine levels [20]. According to the feed additives list in China, eight *Lactobacillus* spp. can be used as feed additives in pig production, including *L. acidophilus*, *L. casei*, *L. delbrueckii* subsp. *Lactis*, *L. plantarum*, *L. reuteri*, *L. cellobiose*, *L. fermentans* and LDB (http://www.moa.gov.cn/nybgb/2014/dyq/201712/t20171219_6104350.htm, accessed on 10 May 2022). Most of them are used as an alternative to antibiotics, including *L. reuteri*, *L. fermentums*, *L. acidophilus*, and *L. salivarius* [21], *L. casei* [22] and *L. plantarum* [23]. According to the Chinese Center of Industrial Culture Collection, the biological hazard of LDB is level four, which means low risk, low pathogenicity, less chance of laboratory infection and no cause of human or animal disease (http://www.china-cicc.org/, accessed on 10 May 2022). LDB is usually used to produce probiotic health food [24]. LDB can inhibit *Escherichia coli* [25] and *Helicobacter pylori* infections [26]. LDB can eliminate *Clostridium difficile*-mediated cytotoxicity and reduce *C. difficile* colonization in colorectal cells [27]. However, few studies have addressed LDB as an antibiotic replacement in pigs.

It is known that bacteria are usually located in the digestive tract, especially in the colon, rectum and cecum. The rectum microbiota forms an extraordinarily complex system that plays a key role in animal physiology and health, including host nutrient metabolism and regulation of carbohydrate metabolism [28]. Microbiota comprises diverse bacteria and other microorganisms, whose abundance is influenced by the host’s genetics [29], age [30], disease status [31] and environmental factors. Previous studies have shown that 16S rRNA technology is suitable for exploring the rectum microbiota [32,33]. Using 16S rRNA technology, Wang et al. reported that the *L. reuteri* effectively reduced *E. coli* in pigs [34]. In addition, Xu et al. showed that *Saccharomyces cerevisiae* regulated the abundance of *Enterococcus*, *Succinivibrio* and *Ruminococcus*, among others [35]. The GIT is where major nutrient metabolism and absorption occurs, and since bacteria metabolize the nutrients, the fecal metabolites become increasingly complex as the diet changes.

Metabolomics is an emerging omics technology that explains differences at the metabolic level and is suitable for identifying fecal biomarkers [36]. Mao et al. illustrated (using metabolomics technology) that *L. rhamnosus GG* substantially increased the concentrations of caprylic acid, 1-mono-olein, erythritol and ethanolamine [37]. Of note, there is a strong association between GIT microbiota and metabolites and 16S rRNA technology, coupled with metabolomics, is able to explain the link between gut microbiota and metabolites. Using 16S rRNA and metabolomics technology, Liang et al. revealed that a diet supplemented with *Clostridium. butyricum* changed 22 metabolites and specific microbiota (such as *Oscillospira*, *Ruminococcaceae_NK4A214_group* and *Megasphaera*) in pigs [38]. However, 16S rRNA technology combined with metabolomics has not yet been applied to LDB in pigs.

The microbiota changes with age in pigs, especially in piglets [39]. According to Wang et al., the microbiota in the growing-finishing stage is relatively stable and sex does not significantly affect swine GIT microbiota [40]. Han et al. reported that sows in the growing-finishing stage (93 d and 147 d) had a stable intestinal environment [41]. These studies suggest that growing-finishing pigs are suitable for analyzing the possibility of replacing antibiotics with probiotics.

In the current study, female growing-finishing pigs were used. 16S rRNA technology was implemented to determine bacteria abundance in the microbiota, while metabolomics technology was used to examine metabolite contents in fecal samples of pigs using a liquid chromatography-mass spectrometry-based (LC-MS), non-targeted metabolomics approach. The relationship between the microbiota and metabolites was explored. 16S rRNA technology and metabolomics technology were used to further explain the possibility of using LDB as an antibiotic replacement in pigs.

## 2. Materials and Methods

### 2.1. Animals and Sample Collection

A total of 42 female growing-finishing pigs (Duroc×Landrace×Yorkshire, 59.39 ± 2.29 kg) in the growing-finishing stage were provided by Nanning Xingda Pig Farm (Nanning, China). All pigs were randomly divided into control (G0) and experimental (G1) groups and were raised under similar feeding management regimes. Each group comprised 21 pigs and three replicates, with seven pigs in each replicate. In this study, G0 animals were fed the basal formula diet with 75 mg/kg aureomycin, while G1 animals were fed the basal formula diet supplemented with 3.0 × 10^9^ colony forming units (CFU)/kg LDB. The LDB was provided by the Chinese Center of Industrial Culture Collection (CICC6098). The basal diet was prepared according to the nutritional needs specified by the NRC (1998). The basal dietary formulation and nutrient contents are shown in Table 1 and Table 2, respectively. The experiment lasted 30 days and none of the pigs in G1 received antibiotic treatment during the study period. Individual fecal samples were collected following rectal stimulation on days 0 (D0) and 30 (D30). All fecal samples were immediately snap-frozen in liquid nitrogen and stored at −80 °C in the laboratory. The body weights of pigs were recorded on D0 and D30, and the average daily gain was calculated. The feed intake in all replicates was recorded and the feed efficiency was calculated. All data were statistically analyzed using t-tests in SPSS 19.0 software. Fecal samples of nine pigs from G0 on day 0 (G0D0), G1 on day 0 (G1T0), G0 on day 30 (G0D30) and G1 on day 30 (G1D30) were randomly selected for 16S rRNA amplicon sequencing and untargeted metabolomic analysis. Differences between G0D0 and G1D0 were used to explore the effects on GIT microbes and metabolites. The difference between G0D30 and G1D30 helped examine the impact of LDB on GIT microbes and metabolites.

### 2.2. 16S rRNA Amplicon Sequencing and Analysis

Microbial genomic DNA from pig fecal samples was extracted using the MagPure Stool DNA LQ Kit (Magen, D6358-03, Guangzhou, China) according to the manufacturer’s instructions. The 16S rRNA gene V3–V4 (341F-805R) region was amplified via PCR using 5′-CCTACGGGNGGCWGCAG-3′ and 5′-GACTACHVGGGTATCTAATCC-3′ as the forward and reverse primers, respectively. The Illumina MiSeq platform was used for sequencing at Benagen (Wuhan, China), and 16S rRNA gene sequence exploration was executed using QIIME2 [42]. Amplicon bioinformatic analysis was performed using EasyAmplicon v 1.0 [9]. The VSEARCH parameters used were min_unique_size 35 in dereplication and sintax_cutoff 0.1 in the removal of plastids and non-bacteria [43]. The samples were rarefied to the lowest sequencing depth of 94,170. The principal coordinate analysis (PCoA) was based on the UniFrac binary distance. Using STAMP software, a *p*-value < 0.05 was defined for significantly different microbes. Metagenomic predictions were completed using Phylogenetic Investigation of Communities by Reconstruction of Unobserved States 2 (PICRUSt 2) and summarized by the Kyoto Encyclopedia of Genes and Genomes (KEGG) pathways [44]. The differences in the KEGG pathways were identified by STAMP software using Storey’s FDR multiple test corrections. The organism-level microbiota phenotypes were predicted using BugBase software [45].

### 2.3. Untargeted Metabolomics Study and Analysis

Each sample (100 ± 1 mg) containing 300 µL methanol was added to 1.5 mL Eppendorf (EP) tubes, vortexed for 30 s to precipitate the proteins and then centrifuged (13,000 rpm, 20 min, 4 °C). The supernatant was filtered using a membrane filter (pore size, 0.22 μm) and evaporated using a vacuum concentrator. The resulting dry residues were redissolved in 100 µL acetonitrile-water (3:1) and centrifuged again (13,000 rpm, 20 min, 4 °C). Finally, the liquid supernatant was transferred to sample vials for analysis using an ultraperformance LC-MS (UPLC-MS) system at Guangxi University (Nanning, China). A Dionex liquid chromatography instrument (UltiMate3000, Thermo Fisher Scientific, Waltham, MA, USA), liquid phase pump (HPG-3400 SD, Thermo Fisher Scientific, USA), column temperature box (TCC-3000 SD, Thermo Fisher Scientific, USA), autosampler (WPS-3000SL, Thermo Fisher Scientific, USA), ACQUITY UPLC BEH C18 column (50 mm × 2.1 mm × 1.7 µm), quadrupole electrostatic field orbit trap high-resolution mass spectrometer and Q-Exactive (Thermo Fisher Scientific, USA) heated electrospray (Thermo Fisher Scientific, USA) were used [46]. The scanning mode was full MS and full MS/dd-MS2 (Table 3). The changes of solvents in the gradient elution of UPLC-MS/MS analysis are shown in Table 4. Compound Discover 3.1 software was used for peak extraction, peak alignment, retention time correction, peak area extraction, accurate mass matching (<25 ppm) and secondary spectra matching. The SIMCA-P program (version 14.1, Umetrics, Umea, Sweden) was used to execute unsupervised principal component analysis (PCA) and supervised orthogonal partial least squares-discriminant analysis (OPLS-DA). Significant differential metabolites were screened using the variable importance in projection (VIP) scores (VIP > 1), fold changes (FC > 2 or FC < 0.5) and *p*-values (*p* < 0.05). The Encyclopedia of Genes and Genomes in the Kyoto Protocol (KEGG, http://www.genome.jp/kegg/, accessed on 12 May 2022), human metabolome database (HMDB, https://hmdb.ca/metabolites/, accessed on 12 May 2022) and MetaboAnalyst 5.0 (https://www.metaboanalyst.ca/, accessed on 12 May 2022) were used to identify potential disordered metabolic pathways.

### 2.4. Co-Occurrence Network of GIT Microbiota and Metabolites

The relationship between the relative abundance of significantly different microbes and the relative concentrations of differential metabolites in each sample was calculated using the Spearman correlation function implemented in *R*. The false discovery rate was applied to the *p*-values obtained from Spearman correlations. An adjusted *p*-value < 0.05 and absolute value > 0.6 were defined as a significant relationship pair. Cytoscape_v3.8.2 was used to construct the co-occurrence network between the GIT microbiota and metabolite significance relationship pair.

## 3. Results

### 3.1. Growth Performance Analysis

The growth indices, including the average daily weight gain, average daily feed intake and feed efficiency, were similar among groups (*p* > 0.05) (Table 5).

### 3.2. Fecal Microbiota Signatures

**Figure 1:**
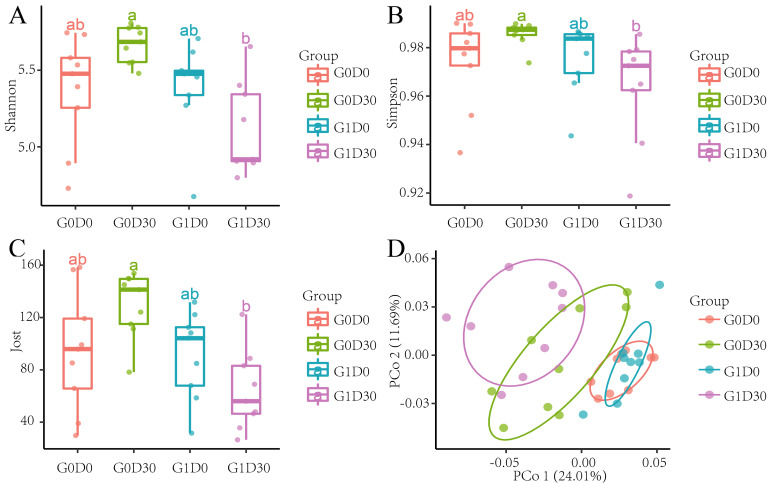
The α-diversity and β-diversity in different groups. (**A**): the Shannon index in different groups, (**B**): the Simpson index in different groups, (**C**): the Jost index in different groups, (**D**): the β-diversity in different groups. Different lowercase letters indicate a significant difference.

**Figure 2:**
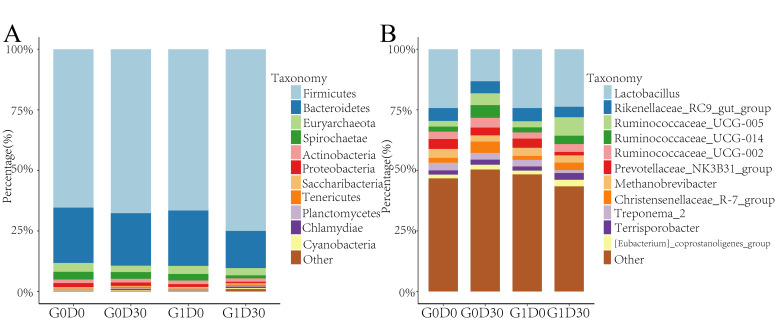
The plot of species composition and differential microbiota. (**A**): the histogram of microbiota at the phylum level, (**B**): the histogram of microbiota at the genus level.

We compared the GIT microbiota differences in G0D0 vs G1D0 and G0D30 vs G1D30 using 16S rRNA amplicon sequencing. After quality control and dereplication, 94, 097 − 263, 187 sequences were obtained. A total of 4534 amplicon sequence variants (ASVs) were identified in our study, and 690 ASVs (22.7%) were found in all samples. After normalization using 94,097 sequences, the bacterial diversity between G0D0, G1D0, G0D30 and G1D30 was compared. The microbial α-diversity indices (Shannon, Simpson and Jost indices) in G0D30 were higher than in G1D30 but not substantially different in G0D0 and G1D0 (Figure 1A–C). Based on the UniFrac binary distance, β-diversity revealed that the microbiota structure differed between G0D30 and G1D30; PCoA1 and PCoA2 explained 24.01% and 11.69% of the variation, respectively (Figure 1D). The relative abundance of bacteria was shown at the phylum (Figure 2A) and genus (Figure 2B) levels. The main bacteria at the phylum level were Firmicutes and Bacteroidetes, accounting for over 82.6%. The average abundance sequences from Firmicutes were 68.65% at the phylum level. Bacteroidetes abundance was 20.69%, while Euryarchaeota, Spirochaetes, Actinobacteria and other bacteria accounted for <2%. A total of 186 genera were identified at the genus level, and the reads from unassigned bacteria were 13.44%. *Lactobacillus* (21.39%), *Rikenellaceae_RC9_gut_group* (5.02%), *Ruminococcaceae_UCG-005* (4.34%), *Ruminococcaceae_UCG-014* (3.24%), *Ruminococcaceae_UCG-002* (3.19%), *Prevotellaceae_NK3B31_group* (3.14%) and *Methanobrevibacter* (3.12%) were the predominantly detected genera.

**Figure 3:**
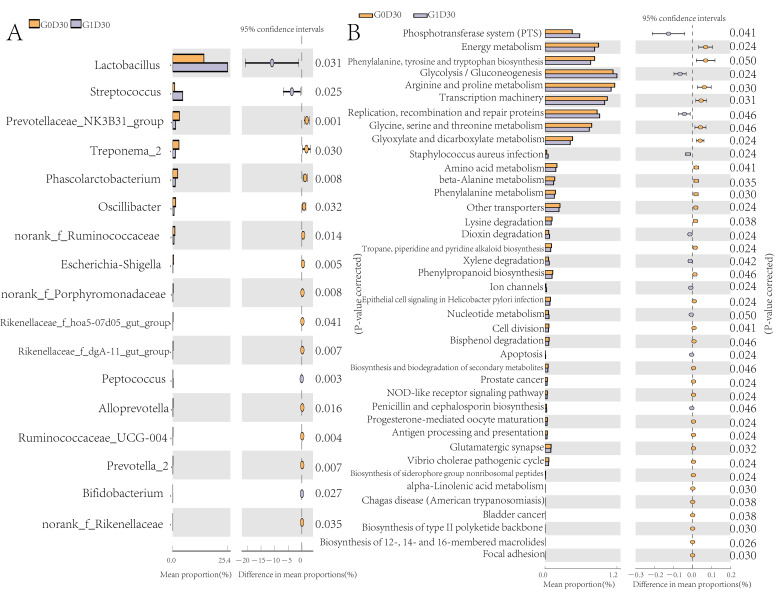
The differential microbiota and differential KEGG pathway. (**A**) the differential genera between G0T30 and G1T30, (**B**) the significantly different pathways between G0D30 and G1D30.

**Figure 4:**
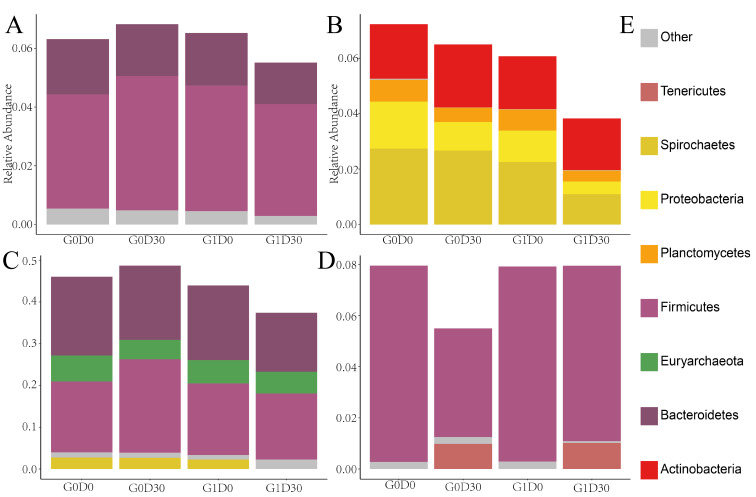
The result of BugBase in different groups. (**A**) The contribution rate in anaerobic. (**B**) The contribution rate in forms biofilms. (**C**) The contribution rate in potentially pathogenic. (**D**) The contribution rate in facultatively anaerobic organisms. (**E**) The legends of (**A**–**D**).

The relative abundance of 17 of the 186 genera, including *norank_f_Rikenellaceae*, *Escherichia-Shigella*, *Rikenellaceae_f_hoa5-07d05_gut_group*, *Rikenellaceae_f_dgA-11_gut_group*, *norank_f_Porphyromonadaceae*, *Ruminococcaceae_UCG-004*, *Treponema_2*, *Prevotellaceae_NK3B31_group*, *Prevotella_2*, *Oscillibacter*, *Alloprevotella*, *Phascolarctobacterium* and *norank_f_Ruminococcaceae*, differed between G0D30 and G1D30 (*p* < 0.05); their abundance was higher in G0D30 than in G1D30, while the abundance of *Lactobacillus*, *Peptococcus*, *Streptococcus* and *Bifidobacterium* in G0D30 was lower than that in G1D30 (Figure 3A). However, there was no substantial difference in microbiota between G0D0 and G1D0. A total of 41 pathways, including amino acid metabolism, energy metabolism and glycolysis/gluconeogenesis, were different between G0D30 and G1D30 (*p* < 0.05) (Figure 3B). The organism-level microbiota phenotypes revealed that anaerobic, biofilm-forming and potentially pathogenic bacteria were more abundant in G0D30 than in G1D30 (*p* < 0.05) (Figure 4A–C) and the abundance of facultative anaerobic organisms in G0D30 was lower than in G1D30 (*p* < 0.05) (Figure 4D).

### 3.3. Fecal Metabolic Signatures

**Figure 5:**
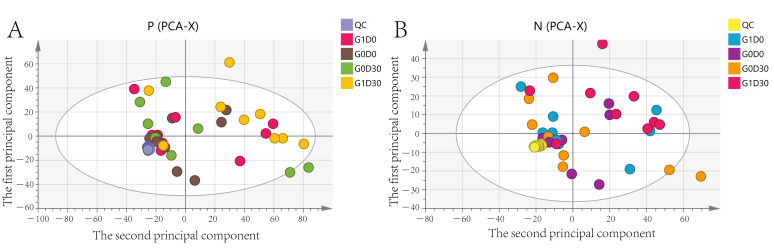
The principal component analysis (PCA) score plot in for different ion modes. (**A**) PCA score plot for the three groups analyzed in the positive ion mode. (**B**) PCA score plot for the three groups analyzed in the negative ion mode.

**Figure 6:**
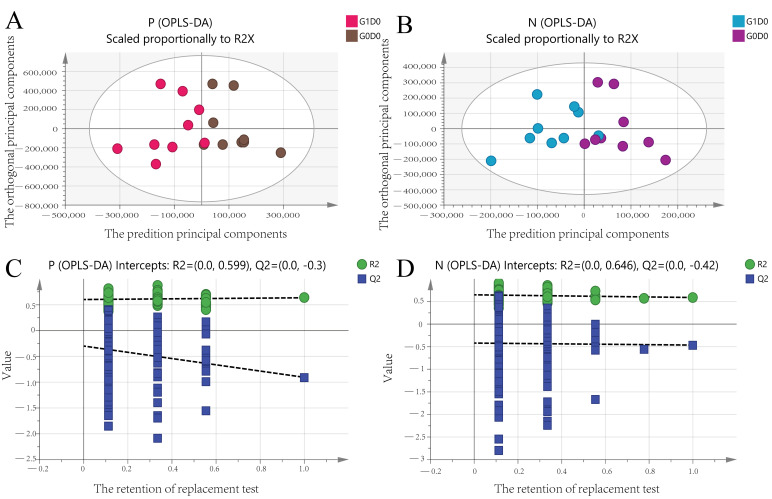
The orthogonal partial least squares discriminant analysis (OPLS-DA) analysis between G0D0 and G1D0. (**A**): The OPLS-DA score plot for the two groups analyzed in the positive ion mode; (**B**): The OPLS-DA score plot for the two groups analyzed in the negative ion mode; (**C**): The OPLS-DA permutation test plot for the two groups analyzed in the positive ion mode; (**D**): The OPLS-DA permutation test plot for the two groups analyzed in the negative ion mode.

**Figure 7:**
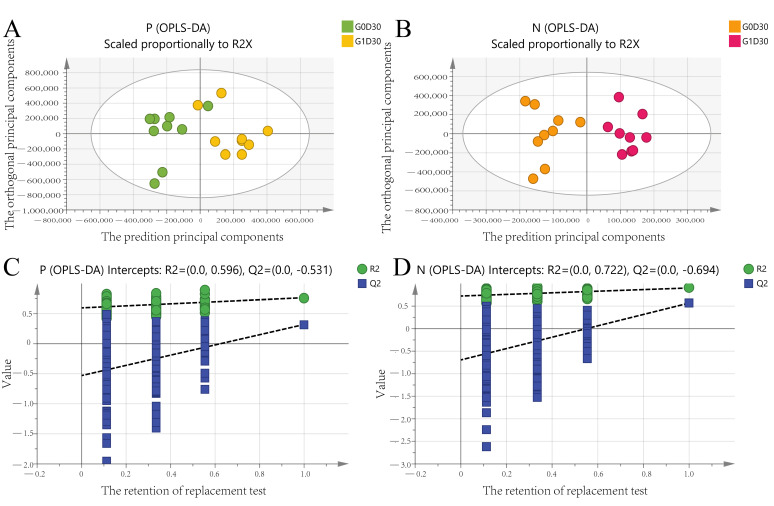
The orthogonal partial least squares discriminant analysis (OPLS-DA) analysis between G0D30 and G1D30. (**A**): The OPLS-DA score plot for the two groups analyzed in the positive ion mode; (**B**): The OPLS-DA score plot for the two groups analyzed in the negative ion mode; (**C**): The OPLS-DA permutation test plot for the two groups analyzed in the positive ion mode; (**D**): The OPLS-DA permutation test plot for the two groups analyzed in the negative ion mode.

**Figure 8:**
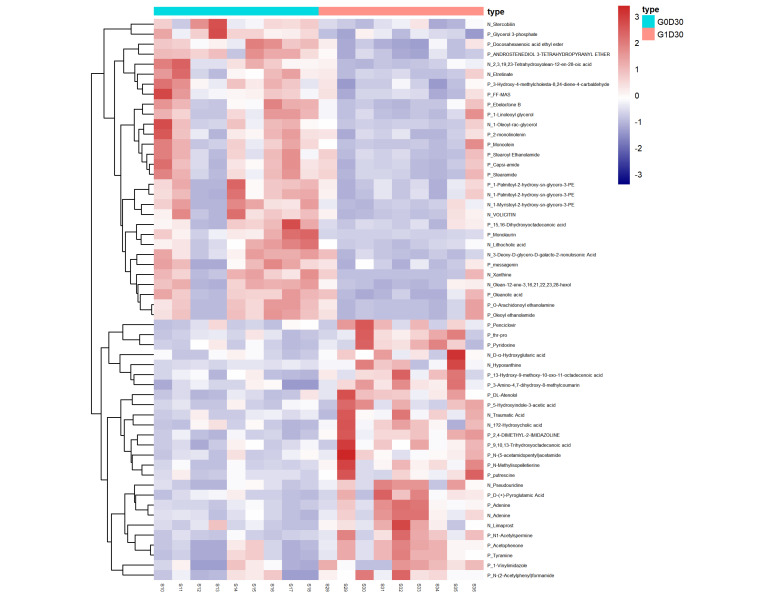
The heatmap of differential metabolites between G0D30 and G1D30.

**Figure 9:**
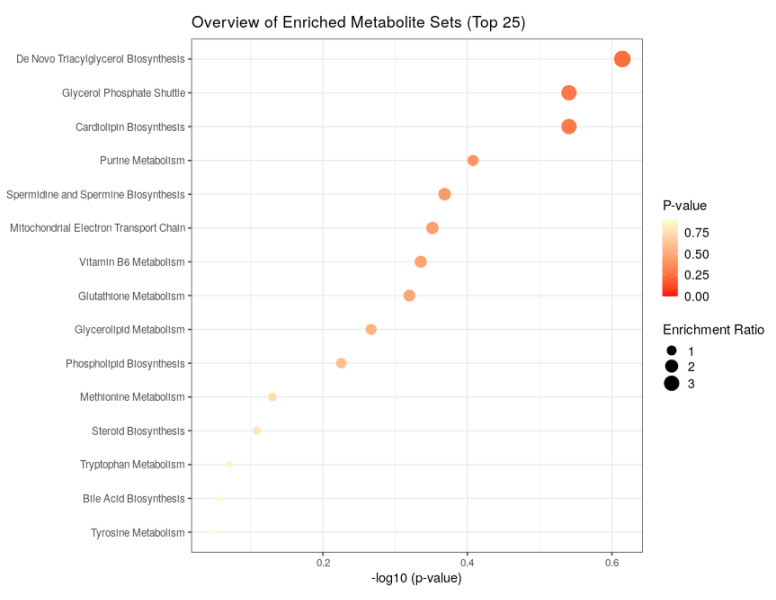
The enriched KEGG in differential metabolites between G0D30 and G1D30.

We explored major differences among metabolites in G0D0 vs. G1D0 and G0D30 vs. G1D30 using untargeted metabolomics in the same samples. A total of 17,941 peaks in the positive and negative ion modes were identified. After filtering, 3488 and 1908 metabolites were matched in the positive and negative ion modes, respectively. The model interpretation rates of X (R2X) were > 0.68 in both the positive and negative ion PCA analyses. Fourteen quality control (QC) samples were obtained under both ion mode conditions (Figure 5A,B). In G0D0 vs. G1D0, the model interpretation rates of Y (R2Y) in the two ion modes were 0.58 and 0.63 (Figure 6A,B), respectively, and the prediction ability (Q2) in the two ion modes was −0.46 and −0.91 (Figure 6C,D), respectively. In G0D30 vs. G1D30, the R2Y and Q2 in the two ion modes were over 0.76 and 0.32 (Figure 7A,B), respectively, and the intercept of the permutation test in the two ion modes was less than −0.53 (Figure 7C,D). Similar metabolites were obtained in the positive and negative ion modes in G0D0 vs. G1D0, respectively (*p* < 0.05). A total of 38 (Table 6) and 18 (Table 7) different metabolites were obtained in the positive and negative ion modes in G0D30 vs. G1D30, respectively (*p* < 0.05) (Figure 8). The 56 differential metabolites in G0D30 vs. G1D30 were enriched in 15 metabolic pathways, including de novo triacylglycerol biosynthesis, glycerol-phosphate shuttle, cardiolipin biosynthesis, purine metabolism, spermidine and spermine biosynthesis, mitochondrial electron transport chain, vitamin B6 metabolism, glutathione metabolism, glycerolipid metabolism, phospholipid biosynthesis, methionine metabolism, steroid biosynthesis, tryptophan metabolism, bile acid biosynthesis and tyrosine metabolic pathway (Figure 9).

### 3.4. Co-Occurrence Network of the Fecal Microbiota and Metabolite Signatures

**Figure 10:**
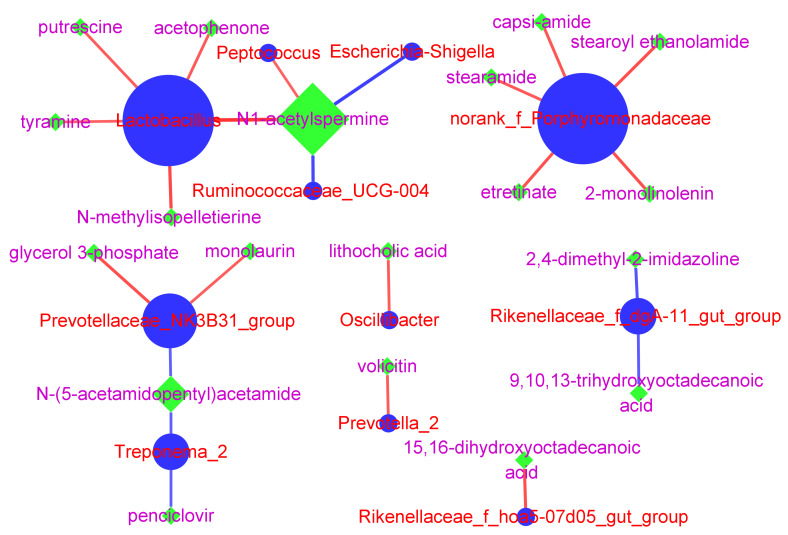
The co-occurrence network between differential microbiota and differential metabolites. The circle shapes were the differential microbiota; the diamond shapes were the differential metabolites. The red lines mean the significant positive correlation; the blue lines mean the significant negative correlation. The size of lines means the correlation size; the size of shapes means the number of networks; the *p* was the positive ion model, and the N was the negative ion model.

The 17 differential microbiota and 56 differential metabolites from G0D30 vs. G1D30 in 36 samples were used to calculate the Spearman correlation coefficients. A total of 437 significant relationship pairs were obtained, including nine microbiota–microbiota, 23 microbiota-metabolite, and 405 metabolite-metabolite relationships (*p* < 0.05). The Spearman correlation coefficients in microbiota-metabolite significant relationship pairs ranged from −0.81 to −0.60 and 0.60 to 0.99. Three major microbiota-metabolite clusters were found (Figure 10). Cluster one in G0D30 vs. G1D30 was composed of *Lactobacillus*, *Peptococcus*, *Ruminococcaceae_UCG-004*, *Escherichia-Shigella*, acetophenone, tyramine, putrescine, N-methylisopelletierine and N1-acetylspermine. Cluster two in G0D30 vs. G1D30 was composed of *norank_f_Porphyromonadaceae*, 2-monolinolenin, capsi-amide, stearoyl ethanolamide, stearamide and etretinate. Cluster three in G0D30 vs. G1D30 comprised *Prevotellaceae_NK3B31_group*, *Treponema_2*, monolaurin, penciclovir, N-(5-acetamidopentyl)acetamide and glycerol 3-phosphate.

## 4. Discussion

Since 2020, the addition of growth-promoting antibiotics in pig diets has been banned throughout China, and microbial feed additives are being considered as an antibiotic replacement. Similar to previous studies [47,48], LDB yielded no adverse effect on feed efficiency. The average daily gain and feed intake in pigs between G0 and G1 did not significantly differ, which may have been related to the short experimental period (30 d) in our study. Nevertheless, these results suggest LDB is a candidate antibiotic replacement in pigs because of the lack of negative effect on growth performance. However, a longer experimental cycle and pigs of different ages are needed to comprehensively elucidate the function of LDB. To determine the possibility of LDB as an antibiotic replacement, we conducted 16S rRNA sequencing and metabolomics. Expectedly, the GIT microbiota and the metabolites were strongly correlated [49,50,51]. The current study revealed major differences between G0D30 and G1D30 in the microbiota and metabolites of the fecal samples from LDB-fed pigs.

The GIT microbiota was dominated by the phyla Firmicutes and Bacteroidetes, which is consistent with the results of prior research [52]. However, in the current study, Firmicutes abundance was increased, and that of Bacteroidetes was decreased in G1D30 compared to G0D30. Interestingly, the contribution of potentially pathogenic forms of *Firmicutes*, *Bacteroidetes* and *Spirochaetes* in G1D30 decreased. The abundance of potentially pathogenic bacteria in G1D30 was substantially reduced, indicating that an LDB-supplemented diet may inhibit the growth of potentially pathogenic bacteria by regulating the GIT function [53]. In G1D30, the abundance of *Streptococcus*, a pathogenic bacterium, was significantly enhanced. *Streptococcus_gallolyticus_*subsp.*_pasteurianus* comprised 83.97% of the *Streptococcus* spp. and was substantially enhanced in G1D30. The high abundance of *Streptococcus_gallolyticus_*subsp.*_pasteurianus* poses a health risk to pigs, especially piglets, because it can cause severe neonatal sepsis and meningitis [54]. The abundance of *Streptococcus_gallolyticus_*subsp.*_pasteurianus* can therefore be controlled should LDB replace antibiotics. Similar to the observations of Bergamaschi et al., the predominant bacterial genus was *Lactobacillus* rather than *Prevotella* [55]. The high abundance of *Lactobacillus* in G1D30 indicates that its presence in the diet is conducive to its abundance in feces [56]. Conversely, the abundance of *Limosilactobacillus* in G1D30 was not increased in the current study, which may be related to the LDB content used in our study. Furthermore, LDB induced a significant increase in *Lactobacillus* abundance and a significant decrease in the abundance of *Treponema_2* and *Prevotellaceae_NK3B31_group* in the top ten most abundant genera. Similar to the results obtained by Sampath et al. [57] and Pupa et al. [14], *Lactobacillus* abundance was increased in pigs fed an LDB-supplement diet in our study. Probiotic supplementation inhibits *Treponema_2* in pig caecal digesta [12]. *L. reuteri* substantially reduced the abundance of *Treponema* sp. in the human mouth [58]. Similar to Xu et al., the abundance of *Prevotellaceae_NK3B31_group* was substantially decreased in pigs administered compound probiotic diets [59]. *Treponema_2* and *Prevotellaceae_NK3B31_group* are Gram-negative bacteria [60] and can produce lipopolysaccharides [61]. Lipopolysaccharides can trigger acute inflammatory responses and the release of inflammatory cytokines and chemokines [62]. Furthermore, as a product of *Lactobacillus* spp., lactic acid plays a key role in antimicrobial, antiviral and immune regulation [63]. The high levels of *Lactobacillus* spp. in the animal GIT can inhibit the abundance of pathogenic bacteria and decrease lipopolysaccharide-induced inflammation [64]. Thus, the LDB diet affected the GIT function by improving the abundance of *Lactobacillus* and *Streptococcus* spp. and decreasing the abundance of *Treponema_2* and *Prevotellaceae_NK3B31_group* spp.

Similar to the β-diversity, the OPLS-DA metabolomics analysis indicated that the LDB diet significantly altered the metabolites. The results of this study suggest that the OPLS-DA model of G0D30 and G1D30 is reliable, stable and devoid of overfitting. The Q2 of G0D0 and G1D0 was less than 0.3, indicating that the OPLS-DA model was unstable, and there were no significant differences between G0D0 and G1D0. A total of 56 metabolites, including four amino acids and derivatives (thr-pro, tyramine, D-(+)-pyroglutamic acid and putrescine), ten fatty acids and derivatives (monoolein, docosahexaenoic acid ethyl ester, oleoyl ethanolamide, 2-monolinolenin, 1-linoleoyl glycerol, 15,16-dihydroxyoctadecanoic acid, traumatic acid, 13-hydroxy-9-methoxy-10-oxo-11-octadecenoic acid, limaprost, N-methylisopelletierine), four monoacylglycerides (monolaurin, monoolein, 2-monolinolenin and 1-linoleoyl glycerol), three purine organic compounds (xanthine, adenine and hypoxanthine), and three cholic acids (stercobilin, lithocholic acid and 1beta-hydroxycholic acid), were identified between G0D30 and G1D30. Oleanolic acid does not inhibit *Lactococcus lactis* but inhibits harmful bacteria [65]. *Lactobacillus plantarum* enhances the concentration of pyridoxine [66] and tyramine [67]. *Lactobacillus fermentation* significantly increases pyroglutamic acid content [68]. The *Latilactobacillus curvatus KP 3–4* increases putrescine concentration in the feces of germ-free mice [69]. Probiotics substantially increase the concentration of 5-hydroxyindole-3-acetic acid [70]. Consistent with Choi et al., the probiotic group contained higher hypoxanthine and lower lithocholic acid contents [71]. Interestingly, the levels of amino acids and their derivatives in G0D30 were substantially lower than those in G1D30, and monoacylglyceride levels in G0D30 were substantially higher than those in G1D30.

The amino acid metabolic pathways for arginine, proline, beta-alanine, glycine, serine and threonine lysine, and phenylalanine were identified in the microbiota. Methionine, tryptophan and tyrosine metabolism pathways were enriched in metabolites. Previous studies have reported that dietary supplementation with *Lactobacillus* spp. influences amino acid metabolism [67,68,69,72]. The energy metabolism was substantially enhanced, and glycolysis/gluconeogenesis pathways were inhibited in G0D30 compared to those in G1D30 in the microbiota. De novo triacylglycerol biosynthesis, the glycerol–phosphate shuttle, mitochondrial electron transport chain, and glycerolipid metabolism pathways were enriched in metabolites. Wang et al. showed that *L. frumenti* promotes porcine energy production [73]. Tang et al. reported that *L. acidophilus NX2-6* enhanced glycolysis and intestinal gluconeogenesis [74]. The LDB diet enhanced the concentration of pyridoxine, tyramine, D-(+)-pyroglutamic acid, hypoxanthine, putrescine and 5-hydroxyindole-3-acetic acid and decreased the concentration of lithocholic acid and regulated the amino acid metabolism and energy production in the pig GIT in a previous study.

*Lactobacillus*, *norank_f_Porphyromonadaceae* and *Prevotellaceae_NK3B31_group* spp. were the core microbiota, and N1-acetylspermine was the core metabolite in the co-occurrence network. Clusters one to three in G0D30 vs. G1D30 contained eight, five and five microbiota–metabolite pairs, respectively. The abundance of *norank_f_Porphyromonadaceae* and *Prevotellaceae_NK3B31_group* in G0D30 was substantially higher than in G1D30. Importantly, *Lactobacillus* and *Prevotellaceae_NK3B31_group* were the most abundant microbiota in our study, and the average abundance of *norank_f_Porphyromonadaceae* in G0D30 and G1D30 was 0.00089 and 0, respectively. Clusters one and three played a more important role in mediating the effects of the LDB on porcine GIT function.

Nonetheless, our study had some limitations. For instance, only female pigs were used, excluding the post-weaning period analysis, and non-inclusion of ileal samples and immunological parameters. However, the results could be useful for the swine industry and public health because of the possible effect of reducing antibiotic use in animal production. Thus, we shall consider genders, growth stages, immunological parameters and ileal samples to comprehensively explain the possibility of using LDB as an antibiotic replacement in our further studies.

## 5. Conclusions

In summary, this study analyzed the different microbiota, metabolite contents and the link between the GIT microbiota and metabolites to explore the possibility of LDB as an antibiotic replacement. Our results revealed that LDB did not adversely affect growth performance. The LDB-supplemented diet increased *Lactobacillus* spp. abundance and the concentration of pyridoxine, tyramine, D-(+)-pyroglutamic acid, hypoxanthine, putrescine and 5-hydroxy indole-3-acetic acid. The LDB diet decreased the abundance of *Prevotellaceae_NK3B31_group* and the concentration of lithocholic acid. The *Lactobacillus* and *Prevotellaceae_NK3B31_group* networks greatly impacted how LDB regulates GIT function in our study. Nonetheless, additional factors such as genders, growth stages and breeds should be considered in our further study to comprehensively explain the mechanism of the LDB-mediated effect on the GIT function of pigs.

## Figures and Tables

**Table 1:** The base formula diet.

Material	Content	Material	Content
Primary corn	43.9%	DL-methionin (98.5%)	0.155%
Flour	6%	L-Threonin (98.5%)	0.136%
Rice bran meal	7.3%	Antioxidant	0.02%
Milk fat	0.5%	Fragrances (Le Daxiang)	0.005%
Soybean meal	17.8%	Emulsifier (bile acid)	0.035%
Expanded soybean	5%	Phytase (high temperature resistant)	0.015%
Barley (with skin)	15%	Vitamin E (500,000 IU)	0.005%
Calcium hydrogen phosphate	1.12%	Glucose oxidase	0.01%
Stone powder	1.19%	Premixed feed for growing pigs	0.5%
Sodium chloride	0.3%	Choline chloride (50%)	0.1%
Bicarb	0.1%	VitC (capsule)	0.015%
L-lysine hydrochloride (70%)	0.487%	Ruyiruyi (Enterococcus faecium)	0.05%

**Table 2:** The nutrition level of the base formula diet.

Team	Content	Team	Content
DE	3.17(Mcal/kg)	ASH	5.81%
CP	16.49%	CF	3.15%
Ca	0.85%	Lys	1.08%
TP	0.66%	Met	0.42%
AP	0.35%	Cys	0.28%
EE	5.75%	Thr	0.75%

**Table 3:** Instrument operation program of UPLC-MS/MS analysis.

Item	Parameter	Item	Parameter
column temperature	30 °C	mass range	80 *m*/*z* to 1200 *m*/*z*
autosampler temperature	4 °C	sheath gas flow rate	30 psi
Sample injection volume	2 µL	auxiliary gas flow rate	10 psi
Mobile phase A	water plus 0.1% formic acid	transmission Capillary temperature	320 °C
Mobile phase B	Methanol	primary scan resolutions	70,000
spray voltage	3.0 kV	secondary scan resolutions	17,500

**Table 4:** Changes of solvents in the gradient elution of UPLC-MS/MS analysis.

Time/min	Mobile Phase A/%	Mobile Phase B/%
0~3	95	5
3~6	95~80	5~20
7~20	80~0	20~100
21~23	0	100
23~23.1	0~95	100-5
23~25	95	5

**Table 5:** The growth performance in two groups.

Team	Number	Average Initial Body Weight/Kg	Average Finish Body Weight/Kg	Average Daily Gain/g	Average Daily Feed Intake/Kg	Feed Efficiency
G0	21	59.55 ± 1.45	88.28 ± 2.70	957.59 ± 65.50	2.55 ± 0.22	2.66 ± 0.07
G1	21	59.24 ± 3.31	88.36 ± 2.95	970.63 ± 13.54	2.61 ± 0.29	2.68 ± 0.27

**Table 6:** Information of 38 differential metabolites in positive ion mode between G0T30 and G1T30.

Name	Formula	Molecular Weight (Da)	VIP Value	Fold Change (G0T0/G0T30)	HMDB Number
ebelactone b	C21 H36 O4	352.26	2.12	2.62	HMDB0251673
docosahexaenoic acid ethyl ester	C24 H36 O2	356.27	2.32	2.59	HMDB0251557
monolaurin	C15 H30 O4	274.21	4.59	20.60	HMDB0245396
1-vinylimidazole	C5 H6 N2	94.05	2.68	0.46	HMDB0244017
3-hydroxy-4-methylcholesta-8,24-diene-4-carbaldehyde	C29 H46 O2	426.35	6.10	2.49	HMDB0062387
capsi-amide	C17 H35 N O	269.27	1.21	2.52	HMDB0040940
13-hydroxy-9-methoxy-10-oxo-11-octadecenoic acid	C19 H34 O5	342.24	1.29	0.44	HMDB0040901
stearamide	C18 H37 N O	283.29	1.01	2.54	HMDB0034146
acetophenone	C8 H8 O	120.06	18.60	0.39	HMDB0033910
15,16-dihydroxyoctadecanoic acid	C18 H36 O4	316.26	1.74	2.40	HMDB0031008
N-methylisopelletierine	C9 H17 N O	155.13	1.06	0.24	HMDB0030326
thr-pro	C9 H16 N2 O4	216.11	1.14	0.43	HMDB0029069
penciclovir	C10 H15 N5 O3	253.12	2.10	0.46	HMDB0014444
O-arachidonoyl ethanolamine	C22 H37 N O2	347.28	1.04	2.40	HMDB0013655
1-linoleoyl glycerol	C21 H38 O4	354.28	5.18	2.40	HMDB0011568
monoolein	C21 H40 O4	356.29	5.01	2.71	HMDB0011567
2-monolinolenin	C21 H36 O4	352.26	2.18	2.42	HMDB0011540
oleoyl ethanolamide	C20 H39 N O2	325.30	1.21	2.42	HMDB0002088
DL-atenolol	C14 H22 N2 O3	266.16	1.59	0.23	HMDB0001924
putrescine	C4 H12 N2	88.10	1.81	0.25	HMDB0001414
N1-acetylspermine	C12 H28 N4 O	244.23	1.31	0.20	HMDB0001186
ff-mas	C29 H46 O	410.35	2.18	2.44	HMDB0001023
5-hydroxyindole-3-acetic acid	C10 H9 N O3	191.06	2.43	0.24	HMDB0000763
tyramine	C8 H11 N O	137.08	9.46	0.39	HMDB0000306
D-(+)-pyroglutamic acid	C5 H7 N O3	129.04	3.03	0.30	HMDB0000267
pyridoxine	C8 H11 N O3	169.07	1.25	0.47	HMDB0000239
glycerol 3-phosphate	C3 H9 O6 P	172.01	1.05	2.41	HMDB0000126
adenine	C5 H5 N5	135.05	3.00	0.29	HMDB0000034
androstenediol 3-tetrahydropyranyl ether	C24 H38 O3	374.28	5.40	2.89	-
1-palmitoyl-2-hydroxy-sn-glycero-3-pe	C21 H44 N O7 P	453.28	1.14	2.33	-
N-(2-acetylphenyl) formamide	C9 H9 N O2	163.06	8.38	0.46	-
9,10,13-trihydroxyoctadecanoic acid	C18 H36 O5	332.26	1.03	0.45	-
3-amino-4,7-dihydroxy-8-methylcoumarin	C10 H9 N O4	207.05	1.80	0.36	-
2,4-dimethyl-2-imidazoline	C5 H10 N2	98.08	1.55	0.36	-
N-(5-acetamidopentyl) acetamide	C9 H18 N2 O2	186.14	1.20	0.33	-
stearoyl ethanolamide	C20 H41 N O2	309.30	1.08	2.48	-
messagenin	C29 H48 O3	444.36	4.42	2.19	-
oleanolic acid	C30 H48 O3	438.35	1.07	2.18	-

**Table 7:** Information of 18 differential metabolites in negative ion mode between G0T30 and G1T30.

**Name**	**Formula**	**NW (Da)**	**VIP**	**FC (G0T0/G0T30)**	**HMDB**
limaprost	C22 H36 O5	380.26	1.79	0.38	HMDB0254093
etretinate	C23 H30 O3	354.22	2.11	2.93	HMDB0252122
stercobilin	C33 H46 N4 O6	594.34	1.16	2.40	HMDB0240259
2,3,19,23-tetrahydroxyolean-12-en-28-oic acid	C30 H48 O6	504.34	1.84	2.54	HMDB0034501
1-oleoyl-rac-glycerol	C21 H40 O4	356.29	1.39	4.14	HMDB0011567
traumatic acid	C12 H20 O4	228.14	1.00	0.44	HMDB0000933
pseudouridine	C9 H12 N2 O6	244.07	2.10	0.45	HMDB0000767
lithocholic acid	C24 H40 O3	376.30	19.14	2.71	HMDB0000761
d-α-hydroxyglutaric acid	C5 H8 O5	148.04	3.26	0.40	HMDB0000428
3-deoxy-d-glycero-d-galacto-2-nonulosonic acid	C9 H16 O9	268.08	3.22	3.80	HMDB0000425
1-hydroxycholic acid	C24 H40 O6	424.28	2.41	0.48	HMDB0000307
xanthine	C5 H4 N4 O2	152.03	6.65	3.54	HMDB0000292
hypoxanthine	C5 H4 N4 O	136.04	4.30	0.29	HMDB0000157
adenine	C5 H5 N5	135.05	1.78	0.35	HMDB0000034
1-myristoyl-2-hydroxy-sn-glycero-3-pe	C19 H40 N O7 P	425.25	1.35	2.60	-
1-palmitoyl-2-hydroxy-sn-glycero-3-pe	C21 H44 N O7 P	453.29	2.81	2.21	-
olean-12-ene-3,16,21,22,23,28-hexol	C30 H50 O6	506.36	2.01	2.40	-
volicitin	C23 H38 N2 O5	422.28	1.19	2.34	-

## Data Availability

The original contributions presented in the study are publicly available. This data can be found here: 10.6084/m9.figshare.19759264.
